# Diversity of uropathogens and their antibiotic resistance among diabetic patients presented to MTI-Lady Reading Hospital, Peshawar

**DOI:** 10.12669/pjms.40.5.8275

**Published:** 2024

**Authors:** Adeela Masood, Muhammad Bilal, Salim Badshah, Yaseen Khan

**Affiliations:** 1 Adeela Masood, MBBS. Medical Officer, MTI Lady Reading Hospital, Peshawar, Pakistan; 2 Muhammad Bilal, MBBS, FCPS, MRCPI, FRCPI, CHPE, CHR, MTI Lady Reading Hospital, Peshawar, Pakistan; 3 Salim Badshah, FCPS. Medical Officer, Health Department, Khyber Pakhtunkhwa, Pakistan; 4 Yaseen Khan, MBBS, FCPS, MRCPI, FRCPI, Professor of Medicine, MTI Lady Reading Hospital, Peshawar, Pakistan

**Keywords:** Antibiotics, Uropathogens, Sensitivity, Bacterial resistance, UTI, Diabetes mellitus

## Abstract

**Objective:**

Our objective was to quantify the number of various bacteria that frequently cause UTI in diabetes patients as well as to gauge their susceptibility and resistance to antibiotics.

**Method:**

A cross-sectional study was conducted at the Internal Medicine Ward of Lady Reading Hospital, Peshawar, Pakistan from June 2021 to December 2021, Patients with confirmed diabetes were included in the study; however, participants receiving antimicrobial medications for a maximum of 14 days were excluded from the study. Resistance of *Escherichia coli, Candida, Pseudomonas, E. faecalis, Klebsiella, P. mirabilis and Staphylococcus* was asssessed using ciprofloxac, ceftazidime and meropenem.

**Results:**

The findings highlighted the the prevalence of *Escherichia coli* in 38.8% of patients, Candida in 19% of patients, *Enterococcus faecalis* in 11.8% of patients, Pseudomonas in 10%, Klebsiella in 9.5% patients, *Proteus mirabilis* 6.2% patients and Staphylococcus was found in 5.2% patients. According to the overall sensitivity and resistance of antibiotics in microorganisms, Meropenem showed 89.6% sensitivity and 10.4% resistance. Ciprofloxacin showed 38.9% sensitivity and 61.1% resistance and ceftazidime showed 22.7 sensitivity and 77.3% resistance.

**Conclusion:**

UTIs were very common in diabetes patients, and *Escherichia coli* was the most common uropathogen found. Compared to male patients, more female patients had infections. The uropathogens showed a significant degree of resistance to ceftizidime and ciprofloxacin.

## INTRODUCTION

The most frequent bacterial infections are urinary tract infections, which account for almost seven million hospital visits and one million ER visits. These infections cause 100,000 hospitalizations of patients with diabetes, multiple sclerosis, spinal cord injuries, elderly patients, and catheters, as well as 100,000 hospitalizations of women.^1^ Patients with diabetes are known to be more susceptible to a number of severe and uncommon urinary tract infections (UTIs).^2^ A recent European study found that women with diabetes had a higher prevalence of asymptomatic bacteriuria (26%) compared to those without diabetes (6%).^3^ It is advised to give diabetic patients extra consideration, particularly when it comes to the treatment of bacterial UTIs, as they are at an increased risk of developing UTIs.^4^

UTI is linked to a number of risk factors, including age, sexual activity, length of diabetes, inadequate glycemic control, and complications from diabetes.^5^ Compared to non-diabetic patients, patients with comorbid illnesses such as diabetes have a higher incidence of urinary tract infections. This is likely because of changes in the genitourinary system, a compromised immune system, altered bacterial adhesion to the uroepithelium, abnormalities in the Tamm-Horsfall Protein (THP), granulocyte dysfunction, the presence of diabetic cystopathy, and microvascular disease in the kidneys.^6^ In addition, women (42.8%) are more likely than men (34.1%) to get a UTI among diabetic patients.^7^,^8^

The majority of unrestricted antibiotic usage leads to an increase in resistance among uropathogens in both community and healthcare settings, and treatment of UTI patients is frequently initiated empirically.^9^,^10^ For appropriate UTI therapeutic interventions, local data regarding the antibiotic resistance of uropathogens should be available. The study’s objective was to assess the range of uropathogens and their profiles of antibiotic resistance in a group of patients with and without diabetes.

## METHOD

In this cross-sectional investigation conducted from from June 2021 to December 2021. Two hundred eleven patients with confirmed diabetes were included. 50% as the anticipated prevalence of UTIs was used to calculate the sample size. The formula n=z²*p (1-p)/d² was used to determine the minimal sample size.^11^ Where n is the necessary sample size, z is the normal deviation (= 1.96), which corresponds to the 95% confidence interval, p is the percentage of the target population with the desired features (0.5 = 50%) and d is degrees of freedom (= 0.05). Patients with confirmed diabetes were included in the study; however, participants receiving antimicrobial medications for a maximum of 14 days were excluded. A self-administered questionnaire was utilized to gather data on demographic and socioeconomic characteristics after each patient gave their informed consent.

### Ethical Approval

The study was approved by ethics and scientific committee (Reference# 543/LRH/MTI; Date 27/07/2020).

Fasting blood glucose, random blood glucose, urine routine examination (dipstick and microscopy), urine culture and sensitivity on urine culture media (McConkey agar, cystine lactose electrolyte deficient medium) were performed. All the data were recorded on predesigned proforma for subsequent analysis. Data analyses were done by using IBM SPSS version 20. Microorganisms and resistance/sensitivity to different drugs were stratified according to different age groups, gender and presenting symptoms to see the role of effect modifiers.

## RESULTS

The study included a total of 211 diabetic patients presented with UTI. The mean age of the patients was 43.86±10.98 years. According to the prevalence of microorganisms, Escherichia coli was found in 81 (38.8%) patients, Candida in 40 (19%) patients, Enterococcus faecalis in 25 (11.8%) patients, Pseudomonas in 21 (10%) patients, Klebsiella in 20 (9.5%) patients, Proteus mirabilis in 13 (6.2%) patients and Staphylococcus was found in 11 (5.2%) patients (Table-I).

**Table-I:** Frequency of microorganism.

Microorganism	Frequency	Percentage
E.Coli	81	38.4
Candida	40	19.0
Enterococcus faecalis	25	11.8
Pseudomonas	21	10.0
Klebsiella	20	9.5
Proteus mirabilus	13	6.2
Staphylococcus	11	5.2

Total	211	100.0

Meropenem demonstrated an overall antibiotic sensitivity and resistance of 89.6% and 10.4%, respectively. Ceftazidime demonstrated 22.7 sensitivity and 77.3% resistance, whereas ciprofloxacin demonstrated 38.9% sensitivity and 61.1% resistance. (Table-II).

**Table-II:** Sensitivity and resistance of antibiotics.

Antibiotic	Sensitivity (%)	Resistance (%)
Meropenem	89.6	10.4
Ciprofloxacin	38.9	61.1
Ceftazidime	22.7	77.3

In uropathogen sensitivity testing, *Escherichia coli* showed a relatively high level of resistance to ceftazidime (82.7%), moderate resistance to ciprofloxacin (55.6%), and low resistance to meropenem (11.1%). Candida exhibited a high level of resistance to ceftazidime (85%) and ciprofloxacin (65%), and low resistance to meropenem (7.5%). Pseudomonas showed similar patterns of resistance to ceftazidime (81%) and ciprofloxacin (61.9%) and meropenem (4.8%). *E. faecalis* exhibited a high level of resistance to ciprofloxacin (68%), moderate resistance to ceftazidime (52%) and and low resistance to meropenem (8%). Klebsiella exhibited a high level of resistance to ceftazidime (65%) and ciprofloxacin (60%) and and low resistance to meropenem (20%). *P. mirabilis* showed high level of resistance to ceftazidime (76.9%) and ciprofloxacin (69.2%) and low resistance to meropenem (7.7%). Staphylococcus showed highl level of resistance to ciprofloxacin (63.6%) and low resistance to ceftazidime (18.2%) and meropenem (18.2%). (Table-III)

**Table-III:** Microorganisms and their sensitivity and resistance to antibiotics.

	Meropenem		Ciprofloxacin		Ceftazidime	

Microorganism	Sensitivity N (%)	Resistance N (%)	Sensitivity N (%)	Resistance N (%)	Sensitivity N (%)	Resistance N (%)
E.Coli	72 (88.9)	9 (11.1)	36 (44.4)	45 (55.6)	14 (17.3)	67 (82.7)
Candida	37 (92.5)	3 (7.5)	14 (35.0)	26 (65.0)	6 (15.0)	34 (85.0)
E. faecalis	23 (92.0)	2 (8.0)	8 (32.0)	17 (68.0)	12 (48.0)	13 (52.0)
Pseudomonas	20 (95.2)	1 (4.8)	8 (38.1)	13 (61.9)	4 (19.0)	17 (81.0)
Klebsiella	16 (80.0)	4 (20.0)	8 (40.0)	12 (60.0)	7 (35.0)	13 (65.0)
P. mirabilis	12 (92.3)	1 (7.7)	4 (30.8)	9 (69.2)	3 (23.1)	10 (76.9)
Staphylococcus	9 (81.8)	2 (18.2)	4 (36.4)	7 (63.6)	9 (81.8)	2 (18.2)

## DISCUSSION

Urinary tract infections (UTIs) are the most common bacterial infectious illness in community practice, with a high incidence of morbidity and monetary cost. UTI infections are thought to affect 150 million individuals annually, costing the world economy more than $6 billion.^12^ Both the lower and higher urinary systems can be impacted by lower urinary tract infections. Cystitis, often known as a lower urinary tract infection (UTI), is characterized by symptoms such as suprapubic pain, frequency, and urgency. The likelihood of an upper UTI, which is typical in most UTI patients, is not excluded only because lower UTI symptoms are present.^13^

UTI is more common in females than in males because the female urethra is physically less efficient at preventing bacterial invasion.^14^ The urothelial mucosa’s attachment to the mucopolysaccharide lining and the vaginal tract’s close proximity to the urethra could be the cause. Other significant risk factors for UTI in women include pregnancy and sexual activity.^15^ The normal increase in plasma volume and decrease in urine concentration in pregnant women (70%) results in glycosuria, which promotes bacterial growth in the urine.^16^ In a non-pregnant state, the uterus is similarly located above the bladder; however, in a pregnant state, the enlarged uterus has an impact on the urinary system. Because bacteria can enter the urethra during sexual activity and can be rubbed up the urethra and into the bladder after childbirth, female sexual activity increases the risk of urethra infection.^17^

Our study found that women were more likely than men to present with UTI (69.7% vs. 30.3%), which is in line with other studies that have found that women are more prone to get UTI.^18^ According to this study, *Escherichia coli* was found in 38.8% of urine samples with positive UTI findings and was the most prevalent gram-negative bacterium. This result is consistent with what previous research has shown.^19^ In this investigation, Candida (19%), Enterococcus faecalis (11.8%), Pseudomonas (10%), Klebsiella (9.5%), Proteus mirabilis (6.2%), and Staphylococcus (5.2%) were also identified bacteria from UTI patients. These results are consistent with research that found E coli prevalence to be 34.4%, candida prevalence to be 17.7%, enterococcus faecalis prevalence to be 10.9%, pseudomonas prevalence to be 10.4%, Klebsiella prevalence to be 8.8%, proteus mirabilis prevalence to be 7.3%, and staphylococcus prevalence to be 5.2%.^10^

E. coli and Klebsiella were shown to be the most common uropathogens in UTIs in various parts of the world. The higher incidence of gramme-negative bacteria from the Enterobacteriaceae family causing UTI is due to a variety of reasons, including their adhesion to the uroepithelium. In addition, adhesins, pili, fimbriae, and the P-1 blood group phenotypic receptor allow them to infiltrate the urogenital mucosa.^20^ Meropenem used in this study was found to be the most sensitive drug against all isolated uropathogens showing 89.6% overall sensitivity. Meropenem was found the most effective against all uropathogens, E. coli (88.9%), Candida (92.5%), Enterococcus faecalis (92%), Pseudomonas (95.2%), Klebsiella (80%), Proteus mirabilis (92.3%) and Staphylococcus (81.8%). The results of this study on antibiotic susceptibility match those of other research.^18^ Meropenem was found to be 95.8% sensitive against extended-spectrum lactamase-producing E. coli in research. Meropenem was followed by amikacin (93.7%) and imipenem (91.71%).^21^

### Suggestions

The results of this study suggest that future research should concentrate on the root causes of resistance to discover answers to this serious issue as well as the use of health education to discourage medication use.

## CONCLUSION

The results of our investigation showed that UTIs were very common in diabetes patients, and *Escherichia coli* was the most common uropathogen found. Compared to male patients, more female patients had infections. The uropathogens showed a significant degree of resistance to ceftizidime and ciprofloxacin.

### Authors Contribution:

**AM** and **MB:** Substantial contributions to conception and design, acquisition of data, analysis and interpretation of data.

**SB** and **AM:** Drafting the article and revising it critically for important intellectual content.

**Yk** and **MB:** Final approval of the version to be published.

**MB:** Agreement to be accountable for all aspects of the work in ensuring that questions related to the accuracy and integrity of any part of the work are appropriately investigated and resolved.
